# Policy-Relevant Research and Media Communication

**DOI:** 10.1093/geroni/igaa057.2384

**Published:** 2020-12-16

**Authors:** Pamela Herd

**Affiliations:** Georgetown University, Washington, District of Columbia, United States

## Abstract

The second speaker is Dr. Pamela Herd, Professor of Public Policy at Georgetown University. Dr. Herd will discuss her approach to conducting innovative and impactful policy-relevant research, as well as her experience communicating research to policymakers and the public through op-eds and other forms of media. Dr. Herd’s research focuses on inequality and how it intersects with health, aging, and policy. She also has expertise in survey methods and administration. Her most recent book, Administrative Burden, was reviewed in the New York Review of Books. She has also published editorials in venues such as the New York Times and the Washington Post, as well as podcasts, including the Weeds, produced by Vox media.

